# Evaluating the Long-Term Efficacy of Acupuncture Therapy for Subacute Poststroke Aphasia: Study Protocol for a Randomized, Blinded, Controlled, Multicentre Trial

**DOI:** 10.1155/2021/8880590

**Published:** 2021-02-12

**Authors:** Xiaolin Li, Ying Gao, Chi Zhang, Qingsu Zhang, Xiyan Xin, Zhongjian Tan, Binlong Zhang, Ruiwen Fan, Xing Huang, Minjie Xu, Xin Shu, Heming Yan, Changming Li, Qiao Kong, Shuren Li, Jingling Chang

**Affiliations:** ^1^Department of Neurology, Dongzhimen Hospital, Beijing University of Chinese Medicine, Beijing 100700, China; ^2^Institute for Brain Disorders, Beijing University of Chinese Medicine, Beijing 100700, China; ^3^Hearing and Speech Rehabilitation Department, China Rehabilitation Research Center, Beijing 100068, China; ^4^Traditional Chinese Medicine Department, Peking University Third Hospital, Beijing 100191, China; ^5^Guang'anmen Hospital, China Academy of Chinese Medical Sciences, Beijing 100053, China; ^6^Division of Nuclear Medicine, Department of Biomedical Imaging and Image-guided Therapy, Medical University of Vienna, Vienna, Austria

## Abstract

**Background:**

Poststroke aphasia (PSA) is a disabling condition that decreases the quality of life, and the duration of the disease harms the quality of life of PSA patients. Acupuncture has been widely employed for PSA. There is some evidence for the immediate treatment efficacy of acupuncture for PSA; however, long-term results after acupuncture may be poorer.

**Methods:**

This is a multicentre, randomized, blinded, nonacupoint (NA) acupuncture controlled, multimodal neuroimaging clinical trial. A total of 48 subjects with subacute PSA will be randomly assigned to an acupoint group or an NA control group. The acupoint group will receive acupuncture with normal needling at DU20, EX-HN1, HT5, GB39, EX-HN12, EX-HN13, and CV23. The NA control group will receive acupuncture in locations not corresponding to acupuncture points as sham acupoints. Both groups will receive identical speech and language therapy thrice a week for four weeks. The primary outcome will be the change in the aphasia quotient (AQ) score measured by the Western Aphasia Battery (WAB) test during the 12th week after randomization. Participants will be blindly assessed at prerandomization (baseline) and 4 weeks, 12 weeks, and 24 weeks after randomization. The secondary outcomes include the Boston Diagnostic Aphasia Examination (BDAE) score, the Disease Prognosis Scale score for ischaemic stroke, etc. Magnetic resonance imaging (MRI) and electroencephalogram (EEG) will also be performed at 4-time intervals as secondary outcomes. All scores and image evaluations will be taken at the same point as the linguistic evaluation. The multilevel evaluation technique will be used to assess the long-term efficacy of acupuncture therapy. MRI scans and EEG will be used to assess acupuncture-related neuroplasticity changes. *Discussion*. The results from our trial will help to supply evidence for the long-term acupuncture effects for PSA over a long follow-up period. It will provide valuable information for future studies in the field of PSA treatment. The trial was registered at the Chinese Clinical Trial Registry on 16 March 2020 (ChiCTR2000030879).

## 1. Introduction

Stroke is a leading cause of mortality and disability globally [1]. Poststroke aphasia (PSA) is one of the most devastating symptoms in stroke survivors [2], who rarely spontaneously recover in the ensuing time. Approximately 30% of stroke patients suffer from aphasia [3], 50% of stroke survivors are still aphasic one year after stroke, and residual symptoms may persist for many years [4]. It can impact an individual's ability to speak, comprehend spoken language, read, and write [5]. Basic requirements of daily life that rely on communication are affected, and social participation can be dramatically impaired. A large-scale survey investigated the relationship between the presence or absence of 75 diseases and the quality of life scores. The highest negative correlation is aphasia [6]. Aphasia rehabilitation has been listed as one of the top 10 research priorities related to life after aphasia [2]. Patients with PSA experience longer hospitalization stays and need more healthcare support, so studying the long-term curative effect of acupuncture treatment on PSA is conducive to maximizing of medical resources.

A review of clinical trials for PSA over the past 5 years revealed that a multitude of interventions can be beneficial in improving language and functional outcomes for patients with PSA [5], with the majority of high-quality clinical research focusing on the chronic phase of aphasia [7–9]. Language disorders are diverse and can change over time. The clinical symptoms may be different in the subacute phase as well as in the chronic phase [10]. Only a few randomized studies (*n* = 12–30) have examined the efficacy of PSA treatment in the subacute phase [11–13]. Research on the subacute period is therefore relatively scarce.

Effective therapies focusing on improving speech and language in patients with PSA are essential. At present, considerable evidence has suggested that treatment with speech and language therapy (SLT) is effective in improving communication and quality of life in individuals with aphasia. These studies have provided evidence of the effectiveness of SLT for people with aphasia following stroke in terms of improved functional communication, reading, writing, and expressive language compared with no therapy [14]; however, the effect sizes of SLT are moderate, potentially reflecting a physiological limit of training-induced progress, the treatment is costly, and progress is often slow. One of the studies also stated that SLT for more than 2 hours a day provided no added value on PSA [15]. It is certain that the various stages of PSA are associated with varying degrees of language recovery, but recovery requires a large number of therapy sessions. It remains a challenge to optimize the effect of aphasia therapy. As a result, an increasing amount of research has been devoted to alternative methods to improve the effectiveness of aphasia treatment, generally by increasing the total amount of treatment achieved [16].

Acupuncture is an easy-to-use, low-cost adjunct to traditional SLT to enhance language outcomes in individuals with PSA. It is one of the main treatments of traditional Chinese medicine (TCM) and has been used for thousands of years. During the past decade, the results of several meta-analyses have concluded that acupuncture after stroke seems to be effective in improving PSA functional communication and language function [17, 18]; however, most of the studies did not assess therapeutic effects over extended periods. For acupuncture treatment, long-term efficacy is considered one of the most important therapeutic effects, which has been suggested by numerous clinical studies [19–25]. It represents the cumulative effect of positive achievements [16]. Long-term efficacy will allow the patient's therapeutic effects to accumulate (“more is better”). Our previous studies on the effects of acupuncture on PSA have shown that participants in the acupuncture group had lower severity than the control group in week 4, but the difference was not significant; however, a significant reduction in PSA severity during weeks 5 to 12 was noted [26]. In addition, we explored the traditional Chinese medical theory and acupuncture technique of this acupuncture programme [27, 28]. Acupuncture has also been shown to have long-term efficacy in chronic pain and tinnitus, which has shown effective therapeutic results [29, 30]. Therefore, we speculate that the long-term therapeutic effect for PSA will also be well maintained; however, the long-term efficacy of acupuncture compared with the placebo effect in patients with PSA has not been investigated.

In terms of the current state of evaluation of aphasia, the requirements for a comprehensive evaluation of PSA have not yet been fully implemented. One of the important means was to evaluate the language characteristics of PSA, which is the major part of the evaluation; however, to date, this single evaluation approach is often insufficient. As such, neuropsychological tests in conjunction with in vivo measures may be more sensitive than neuropsychological tests alone in the assessment of brain structure and electrophysiology [31–33]. We introduce MRI and EEG for evaluation of the objective characteristics of PSA because the multimodal assessment of brain structure and function allows for a more objective evaluation of the recovery characteristics of patients with aphasia. Based on the previous study [34], we conducted a TCM syndrome evaluation of PSA patients and confirmed the relationship between linguistic features and TCM syndrome. Therefore, in this study, we have also introduced the evaluation of TCM syndrome to provide more comprehensive evidence of efficacy based on the combined evaluation of Chinese and Western medicine. For the reasons above, we propose to investigate whether acupuncture treatment on PSA has the value of maintaining long-term efficacy through this randomized controlled trial (RCT) with multimodal evaluation.

We designed an assessor- and participant-blinded RCT with PSA patients. Using NA acupuncture as a control, the trial aims to identify the efficacy of acupuncture by answering two questions: (1) what is the long-term efficacy and reliability of acupuncture as an intervention for PSA compared with the placebo effect? and (2) what interactions will be observed through an integrated evaluation of linguistic features, brain function, and TCM syndrome?

## 2. Methods and Analysis

### 2.1. Study Design

**Figure 1:**
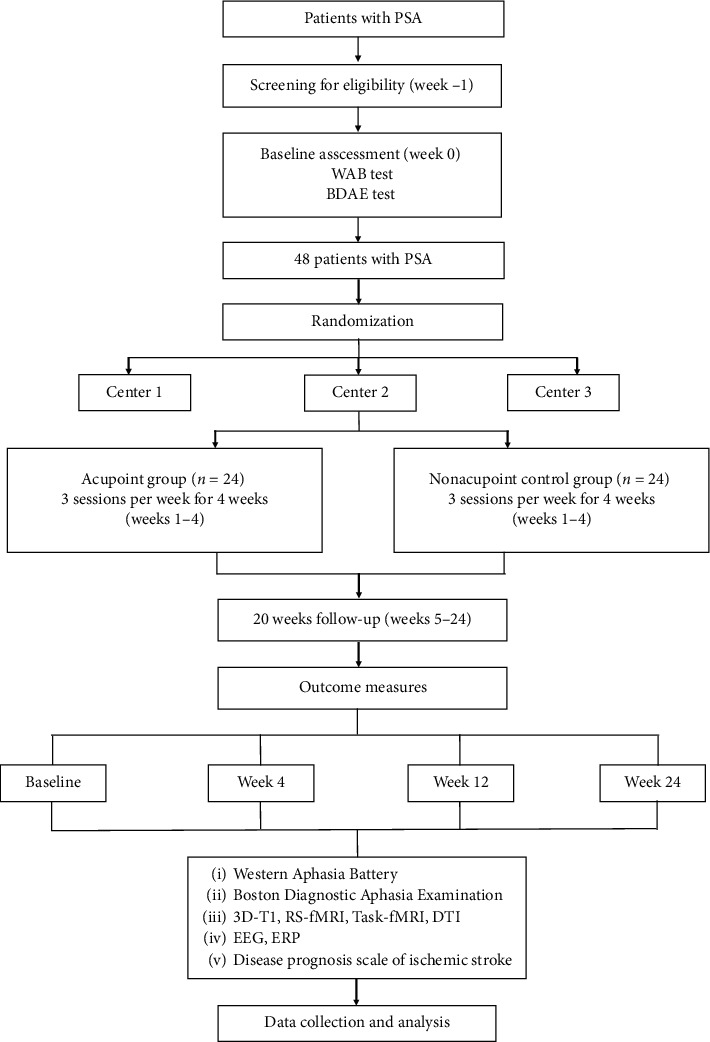
Trial flow chart. WAB, Western Aphasia Battery, BDAE, Boston Diagnostic Aphasia Examination; RS-fMRI, resting-state functional magnetic resonance imaging; Task-fMRI, task functional magnetic resonance imaging; DTI, diffusion tensor imaging; EEG, electroencephalogram; ERP, event-related potential.

This is a multicentre, randomized, assessor- and participant-blinded, NA acupuncture controlled, multimodal neuroimaging clinical trial. It aims to compare the long-term efficacy of the acupoint group and NA control group (in locations not corresponding to acupuncture points). In all groups, participants will receive identical SLT but will be permitted to use medications for the basic internal medicine treatment of stroke. The type, dose, and time of administration of the agent will be recorded in the case report form. The trial design is depicted by the flow diagram in Figure 1. The timeline for study enrolment, intervention, and assessment is illustrated in Table 1. We designed this acupuncture research at full length following the Standard Protocol Items: Recommendations for Interventional Trials (SPIRIT) 2013 statement [35]. The design and reporting of the study will follow the Consolidated Standards of Reporting Trials (CONSORT) statement for nonpharmacological interventions [36] (http://www.consort-statement.org/home/).

### 2.2. Randomization and Blinding

Eligible patients will be randomly assigned to the acupoint group or NA control group with a 1 : 1 ratio. The randomization sequence will be generated by a third-party professional statistician using a computer-generated randomization digital table using SAS V.9.4 software (SAS Institute Inc., North Carolina, USA). An independent assessor will interview the participants and carry out the screening. Random numbers and group assignments will be confirmed immediately through short message service to the practitioners who conduct acupuncture. All participants will be blinded to the types of acupuncture. An independent, blinded assessor who does not know the group assignment will conduct the outcome evaluation after the treatment. The researcher who will oversee the statistical analysis will also be blinded, with the treatment for each group remaining unknown; however, it is impossible to blind practitioners who conduct acupuncture. The practitioner will be forbidden from discussing the type of acupuncture with the participants. We will endeavour to ensure that subjects begin the trial with similar expectations of efficacy by informing them that the provided treatments are effective. In this study, participants, assessors, and statisticians will be blinded to treatment allocation. Participants will receive treatments alone at different times to avoid communication with each other. An eye patch will be applied to patients during the acupuncture treatment. To minimize the unintentional physical cues and bias in this trial, acupuncturists will be required to emulate the same procedure for the nonacupoint control group. In addition, participants will be asked to answer the following question during week 4 to test the blinding effect: “Do you think you have received real acupuncture treatment?” The participants can choose “yes” or “no” as an answer [37, 38]. The percentage of participants who answered “yes” in both groups after the final treatment will be analyzed. If the results show no significant difference in the response to this question between the two groups, they could suggest that the blinding effect is sufficient.

### 2.3. Setting and Recruitment

Participants who meet the inclusion and exclusion criteria will be recruited from the inpatients in the Dongzhimen Hospital affiliated to Beijing University of Chinese Medicine (BUCM), China Rehabilitation Research Center, and Peking University Third Hospital. Participating institutions and the level of the institution are listed in Table 2. The study will be advertised through the Internet and posters in communities and hospitals. The inpatients and potential participants will call an investigator and be prescreened for eligibility and will learn how to participate in this clinical trial through visits or telephone calls to our hospital. During visits to the clinical research centre of Dongzhimen Hospital affiliated to BUCM, China Rehabilitation Research Center, and Peking University Third Hospital, the accessors will explain the study to the patients, who will be asked to sign an informed consent form before voluntary participation. The participants of this study will be selected from the applicants who meet all the inclusion criteria but do not meet any of the exclusion criteria. To facilitate participation in this study, the accessors will properly adjust the evaluation and treatment schedule of the enrolled participants. Therefore, the enrolled participant will be able to complete the treatment and evaluation. Every time the enrolled participant visits, the accessors will inform the participant of the next scheduled visit, and the day before the visit, the accessors will remind the enrolled participant of the schedule by telephone.

### 2.4. Eligibility Criteria

#### 2.4.1. Inclusion Criteria

Participants meeting all of the following criteria will be included in this trial: (1) diagnosed as stroke through computed tomography (CT) or MRI, 1 to 6 months after stroke onset; (2) 30 to 80 years of age (After the expert discussion and the actual situation of our country, the age range has been adjusted), the native language is Chinese, and right-handed; (3) primary school and above education with no serious heart, liver, or kidney diseases; (4) clear consciousness and no cognitive impairment; (5) normal language function before the stroke onset and dominant language dysfunction with mild limb dysfunction; (6) specific aphasia syndrome diagnosed as motor aphasia by the WAB; (7) BDAE score of 2 to 4; and (8) able to cooperate for the 30-minute MRI examination.

#### 2.4.2. Exclusion Criteria

The exclusion criteria are as follows: (1) received pacemaker surgery, coronary intervention, or coronary artery bypass surgery or have other metal products in the body; (2) language dysfunction caused by congenital or childhood diseases; (3) language dysfunction caused by mental disturbance and normal mental retardation; (4) severe dysarthria and hearing impairment; (5) superficial sensation abnormalities in the neurological examination, and (6) participation in other studies.

### 2.5. Interventions

Acupuncture will be performed by registered acupuncturists with over 2 years of experience who will be trained in the standardization of the acupuncture scheme. Only sterile, stainless steel, disposable acupuncture needles (size 0.25 mm × 40 mm, product no. 20182270011; ANDE Acupuncture, Guizhou ANDE Medical Equipment, China) will be used. Both acupuncture and sham acupuncture points will be located in the limbs and head. All the participants will receive 3 treatment sessions per week (alternate days) for 4 consecutive weeks, resulting in a total of 12 sessions. Each treatment will be administered for 30 minutes. The acupuncture and sham acupuncture interventions will be performed by a consensus of acupuncture experts. To ensure strict adherence to the study protocol, the experts will receive training together and use the same techniques. The details of the acupuncture treatment are described in the Standards for Reporting Interventions in Clinical Trials of Acupuncture (STRICTA [39]) checklist in Table 3.

#### 2.5.1. Acupoint Group

**Figure 2:**
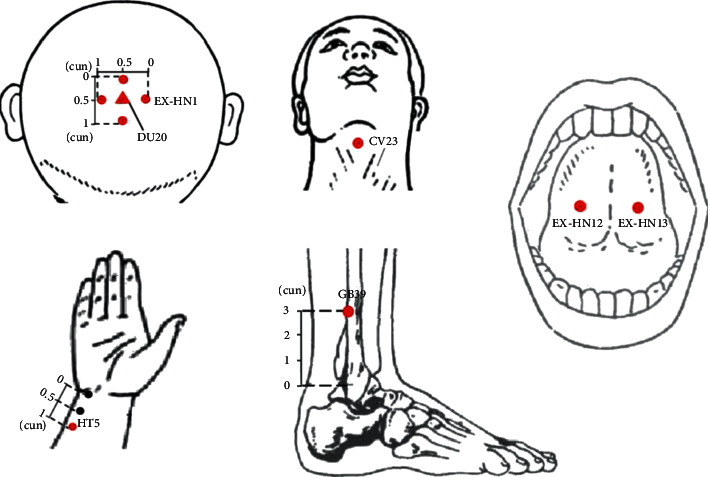
Locations of acupoints.

Participants in the acupoint group will receive acupuncture at bilateral Tongli (HT5) and Xuanzhong (GB39) limb acupuncture points. After skin disinfection, acupuncture needles will be inserted through the skin and approximately 0.5 cun [≈10 mm] into the skin. Needle insertion will follow an angle of 90° in an inferomedial direction for the two points. Following needle insertion, small, equal manipulations of twirling and thrusting will be performed on all needles to reach de qi (a composite of sensations including numbness, distention, soreness, and heaviness that is an important indicator of successful acupuncture treatment), which is believed to be an essential component for acupuncture efficacy. The needles placed in HT5 and GB39 will be manually stimulated every 10 minutes. The acupuncture points on the head are Lianquan (RN23), Jinjin (EX-HN12), Yuye (EX-HN13), Baihui (DU20), and Sishencong (EX-HN1). For RN23, acupuncture needles will be inserted through the skin and approximately 1 cun [≈20 mm] into the skin. Following needle insertion, small, equal manipulations will be performed on the needles to reach numbness. EX-HN12 and EX-HN13 will be quickly inserted for bloodletting. DU20 and EX-HN1 will be inserted through the skin approximately 0.5 cun. Needle insertion will follow an angle of 15° in an inferomedial direction for the two points (for details, see Table 4 and Figure 2).

#### 2.5.2. NA Control Group

**Figure 3:**
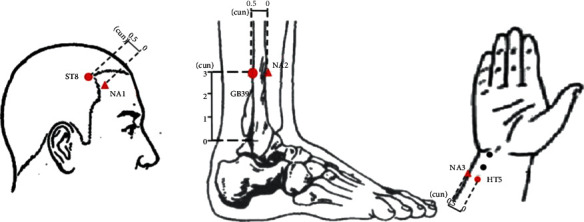
Locations of nonacupoints. NA, nonacupoint.

Participants in the NA control group will receive sham acupuncture with real needles on an NA. NA 1 will be 0.5 cun (≈10 mm) lateral to HT5. NA 2 will be 0.5 cun (≈10 mm) horizontal to GB39, and NA 3 will be 0.5 cun (≈10 mm) lateral to ST8 (for details, see Table 5 and Figure 3). Procedures and other treatment settings will be the same as in the acupoint group but with no needle manipulation for de qi. In both groups, the needles will be retained for 30 minutes for each treatment session. The participants will be treated with acupuncture three times a week, on alternate days, for 4 successive weeks, resulting in a total of 12 sessions for each patient.

#### 2.5.3. Permitted and Prohibited Concomitant Treatments

All the participants will receive identical SLT. The treatment period is 4 weeks (3 treatment sessions per week, alternate days), resulting in a total of 12 sessions. Speech therapy will be performed by an experienced language therapist who received standardized training in language rehabilitation.

The language rehabilitation training method will be as follows: first, the patients will be assessed for their language function and scored, and then a training plan and training principles will be formulated according to the type of aphasia and language ability; the training is progressively followed by targeted strengthening exercises. The main content includes motor training of pronunciation organs, oral pronunciation training, naming, intonation, etc. (1) Mouth shape and voice training: at the beginning of the training, patients will be taught to control their lip and tongue movements through mouth shape and voice control to practice pronunciation. The patients will practice the rhymes and consonants first and then gradually transition to the differentiation of approximate sounds. (2) Use of language training equipment (cell phones, iPads, etc.): patients can use phrases and sentences from daily life to make audio files that are suitable for reading. The patients will practice the phrases first, and then, the sentences. (3) Training the pronunciation muscles: patients with aphasia may have different degrees of wasting atrophy of the pronunciation-related muscles, which results in slurred speech. During training, patients are instructed to practice tonal movements of the tongue and oral muscles to promote accurate pronunciation. (4) Regular check-ups and weakness reinforcement exercises: weaknesses in pronunciation for targeted exercises will be identified, and individual reinforcement training for patients will be provided if necessary.

### 2.6. Sample Size Calculation

No large RCTs have been carried out within the field examining the long-term effects of acupuncture on PSA. Previous studies have used interventions not applicable to the current protocol and have been of varying quality and used small sample sizes. We were, therefore, unable to calculate the prior accurate sample size. Thus, our RCT will support a more accurate sample size estimate and inform a definitive trial examining the long-term effectiveness of acupuncture for aphasia. The study involves the experimental study of functional magnetic resonance imaging (fMRI) and EEG for language tasks, which falls under the scope of experimental psychology. Taking into account individual psychological differences, it is expected that the PSA patients to be included will be divided into 2 groups. The number of patients to be included in the statistics in each group will be 20. Considering a drop-out rate of 20%, the target recruitment number is 48 participants (24 per group).

### 2.7. Multimodal Data Acquisition

#### 2.7.1. MRI Data Acquisition

Patients will undergo brain MRI at prerandomization (baseline) and 4 weeks, 12 weeks, and 24 weeks after randomization. MRI scans will be performed with a 3.0-T MR scanner (Siemens AG, Germany) in the Dongzhimen Hospital affiliated to BUCM. The parameters of the sequences to be employed in this study are provided by the China Association of Brain Imaging. Sagittal structural images will be acquired using a magnetization-prepared rapid gradient-echo three-dimensional (3D) T1-weighted sequence with the following parameters: repetition time (TR)/echo time (TE) = 1900/2.13 ms, flip angle = 9°, inversion time = 1100 ms, resolution = 256 × 256, voxel size: 1.0 × 1.0 × 1.0, 1-mm slice thickness without slice gap.

RS-fMRI and task-fMRI will be performed using an echo-planar imaging (EPI) sequence with the following parameters: TR/TE = 2000/30 ms, flip angle = 90°, resolution = 64 × 64, FOV = 225 × 225, bandwidth = 2520, slice thickness = 3.5 mm with 0.7 mm slice gap, 31 axial slices, and voxel size: 3.5 × 3.5 × 3.5.

Diffusion tensor imaging (DTI) will be acquired with a diffusion-weighted, single-shot, spin-echo, echo-planar imaging sequence that uses 30 directions with *b* = 0 s/mm^2^ and *b* = 1000 s/mm^2^, slice thickness: 2 mm, gap = 0 mm, slices = 65,TR: 11000 ms, TE: 94 ms, matrix: 128 × 128, FOV: 256 × 256, and phase encode direction: *A* >> P.

All scans will be qualitatively reviewed by two radiologists to screen for possible brain lesions or structural abnormalities. fMRI data will be collected during the word-picture judgement task and at rest. fMRI data will be performed with the SPM12 (https://www.fil.ion.ucl.ac.uk/spm/software/spm12/) for MATLAB. Brain activation and connectivity changes will be compared between the two groups.

#### 2.7.2. EEG Data Acquisition

The 64-channel EEG recording and analysis system produced by the Neuroscan of Australia, E-prime 2.0 stimulation display software, scan data analysis software, SYNAMPS EEG amplifier, Fastrak 3D imaging digital instrument, Quick-Cap electrode cap, recording electrode, electrode paste, and Curry multichannel neuroimaging software will be used to collect EEG signals. EEG data in the resting state will be collected for 8 minutes, and event-related potentials (ERPs) will be collected during language task assessment for 8 minutes. The same word-picture judgement task used for the MRI will be applied to ERP collection. Patients will undergo an EEG at a different time on the same day as the MRI scan.

#### 2.7.3. Word-Picture Judgement Task

**Figure 4:**
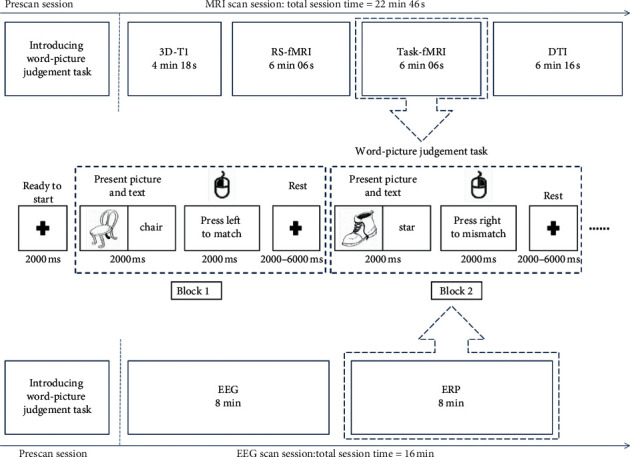
MRI experimental paradigm and an illustrative diagram of the word-picture judgement task. For fMRI, there were 60 blocks. For ERP, 60 blocks. When presenting the task, the word is displayed in Chinese.

The patients will be trained before entering the fMRI scanner. They will complete a practice version of the word-picture judgement task paradigm in the computer. They need to perform the task and reach an accuracy criterion of 90% to ensure that the patients understand how to do the task in the scanner. Patients will view black and white line pictures and Chinese high-frequency nouns. The patients are required to press a mouse button when the picture and the noun appear on a white background. The patients are asked to press the left button if the picture and the noun express the same meaning; otherwise, they should right-click. The stimuli are presented in 60 blocks on a computer using E-prime 2.0. In the ERP experiment, there will be 120 blocks. Trial types within blocks are presented in a pseudorandomized order. During the MRI scanning, all patients are asked to lie quietly in the scanner with their eyes open, trying to avoid systematic thinking, and moving as little as possible. In task-state fMRI scanning, the patients are instructed to maintain a central view and try to not think of other things. During ERP testing, patients are asked to sit in front of the computer, and the surroundings will be kept quiet (Figure 4).

### 2.8. Outcome Measures

#### 2.8.1. Clinical Outcome Assessments

The primary outcome will be the change in the AQ score during the 12th week after randomization. For the assessment of language functioning, the WAB with the subtests spontaneous speech, auditory comprehension, repetition, and naming is included in the test battery. The WAB test will be conducted based on a previous study [40]. Secondary outcomes include four items, the details of which are listed in Table 1.

#### 2.8.2. Neuroplasticity Assessments

In this study, neuroplasticity changes between the two groups will be measured by MRI. Before acupuncture treatment, patients will complete the fMRI scan within 3 days. They will also have a follow-up fMRI scan within 3 days after the completion of their intervention and at 8 weeks and 16 weeks after the intervention (Figure 1). Brain activity and functional connectivity will be assessed under a resting state and a word-picture judgement task. Group differences in white matter integrity will be assessed using DTI.

#### 2.8.3. Assessment of Acupuncture Safety

Acupuncture is considered to be a generally safe procedure [41–43]; however, all adverse events will be recorded in every detail. In this study, acupuncture-related adverse events would mainly refer to broken needles, fainting due to the needling procedure, local infection, hematoma, and other events that can be caused by acupuncture (such as headache, dizziness, and insomnia).

#### 2.8.4. Quality Control

All practitioners will conduct the study according to the standard operating procedure of the study. We will conduct several simulations for our volunteers before the start of the study. During the study, regular meetings will be held to discuss issues raised by researchers or participants, the need to improve protocols, the side effects, and participant recruitment.

### 2.9. Statistical Analysis

#### 2.9.1. Clinical Data Analysis

All statistical analyses will be performed by a statistician from the Clinical Evaluation and Analysis Centre of Dongzhimen Hospital affiliated to BUCM using Statistical Package for the Social Sciences (SPSS) V.22.0. The statistician will be blinded to the allocation of groups. The level of significance is established at *α* < 0.05 with a two-tailed test. The main objective is to compare the change in the AQ score at week 12 from baseline between the acupoint group and the NA control group. The null hypothesis is that the acupoint group will show the same change as the NA control group, while the alternative hypothesis is that the acupoint group shows a greater improvement. Categorical data will be represented by percentages, whereas continuous data will be represented by the average, standard deviation, median, minimum value, and maximum value. For comparison with the baseline, a *t*-test or nonparametric test will be used for continuous data and nonparametric tests for categorical data. For comparison of two independent samples, if the residuals are normally distributed, the analysis of covariance (ANCOVA) will be used for the primary outcome and subgroup analysis stratified by aphasia severity, *t*-tests for other continuous data, and chi-square tests for categorical data; if the residuals are non-normally distributed, a nonparametric test will be used for both the continuous and categorical data. The results of the intention-to-treat (ITT) analysis will be used to assess the validity of the study as a whole. The ITT analysis will collect data from all the participants in this trial, and for those lost to follow-up, the last observation carried forward method will be implemented.

#### 2.9.2. MRI Data Analysis

For imaging, data will be analyzed using the Data Processing & Analysis for (Resting-State) Brain Imaging (DPABI) toolbox [44] performed on MATLAB V.8.6 (MathWorks) to detect any changes in brain function due to acupuncture treatment. Lesion symptom mapping was demarcated on T1-weighted images manually using MRIcroGL (http://www.cabiatl.com/mricrogl/) in native space by neurologists who were blinded to the participants' language scores. DTI images will be analyzed using MATLAB (https://www.mathworks.com/) and FMRIB Software Library (http://www.fmrib.ox.ac.uk/fsl). After data preprocessing, some data-driven approaches will be performed to investigate neuroplasticity between the two groups, such as the amplitude of the low-frequency fluctuation, regional homogeneity, and voxel-wise degree centrality. A two-sample *t*-test will be conducted to investigate the differences in brain regions between the acupoint group or NA control group in the DPABI software. Multiple comparisons will be used to better control for a highly inflated false positivity rate. Pearson's correlation analysis will be performed to examine the association between the fMRI data and clinical variables.

#### 2.9.3. EEG Data Analysis

EEG data will be preprocessed by NeuroScan software, and electrocardiogram, ophthalmic, and myoelectric artefacts will be removed by independent component analysis (ICA). eLORETA software will be used to extract the characteristic indexes of the brain network. The electric current sources in different brain regions will be accurately and intuitively calculated, and the functional network characteristics of the brain in different frequency bands will be analyzed.

#### 2.9.4. ERP Data Analysis

Using the threshold selection method based on a small-world network across different thresholds to build a functional brain network, analyses will be conducted to examine the network topological structure of the brain under the different thresholds, build different threshold brain networks, and use the theory of the complex network diagram analysis method to calculate the weighted aggregation coefficient, weighted characteristic path length, and small-world network features.

## 3. Discussion

PSA has proven to be difficult to treat. There is a degree of spontaneous recovery in the subacute phase, so there are few studies on the subacute phase of PSA. However, the subacute phase also requires treatment. The extant literature shows that acupuncture is probably effective for PSA [18, 45–47]. We intend to evaluate the long-term efficacy of acupuncture in maintaining speech production function. One common problem is the lack of standardization of acupoint selection, needle retention time, number of needles used, needling depth, and needle manipulation in acupuncture research.

From 2006 to now, we attempted to explore the general acupuncture scheme based on syndrome differentiation. In our PSA acupuncture treatment programme, DU20 is located in the area at the top of the head and is particularly closely related to the regulation of brain speech activity. The EX-HN1 are the acupuncture points around the DU20, both of which are used together to benefit the brain and promote speech rehabilitation. The treatment of PSA by EX-HN12 and EX-HN13 bloodletting has a theoretical basis in TCM and unique clinical efficacy. CV23 is mainly used for the treatment of speechlessness, stabbing into the skin towards the root of the tongue, and has the effect of restoring speech. The acupuncture theory of HT5 and GB39 to be used in our study is different from the traditional meaning of these points. FMRI can help us evaluate the changes of needle-related neuroplasticity, so we have conducted fMRI experiments based on this pair of effective points for aphasia. Electroacupuncture at HT5 and GB39 may modulate language and cognition function through a complex network formed by an extensive area of the brain cortex. It is beneficial to the recovery of language function [47]. In previous studies, we have demonstrated that this acupuncture protocol has shown significant improvements in auditory comprehension, reading, and dictation in patients with subacute PSA after 4 weeks of treatment. Therefore, we would like to know more about whether this treatment programme has long-term effects in these areas and observe the association with overall linguistic changes.

Regarding the study design, we developed an assessor- and participant-blinded design to minimize bias. The specific effects are thought to be generated by needling the acupoint with the appropriate manipulation. The nonspecific effects are due to other aspects of the therapy, such as the expectations of the patient, which might influence the treatment outcome. To recognize the specific effects, a placebo is needed. Among acupuncture research, placebo controls for acupuncture studies have been difficult to select. To maximally exclude the placebo effect, rigorous methodological designs are followed. In our study, control conditions involve being punctured with real acupuncture needles at NA locations. Thus, the self-perception of placebo effects in the NA control group is difficult to distinguish from the real acupoints. Additional needle manipulations will not be used in the NA control group. De qi is a characteristic constellation of sensations felt by patients during acupuncture needling. It has been regarded as an important factor related to clinical effects; however, we do not pursue this kind of sensation in the NA control group. By following these methods, participants can be successfully blinded, and the efficacy of acupuncture could be confirmed if the results of the acupoint group prove superior to those of the NA control group.

We will assess patients with PSA on multiple levels, including multiple neuropsychological tests, functional and structural brain alterations, and TCM syndrome evaluation. This multidomain assessment will be used to identify possible biomarkers involved in the effects of acupuncture in PSA.

It is worth mentioning that functional and structural brain alterations will be used as outcome measures. MRI allows for noninvasive evaluations of neural functional changes [48]. fMRI studies use two modalities, task-related and resting-state methods [49]. Task-related fMRI has revealed functional disturbances in individuals with aphasia. MRI may be more sensitive to smaller treatment effects. It can be used as a tool to assess the efficacy of acupuncture in treating aphasia. These analyses may identify whether neural efficiency is improved or the brain connectome is reorganized to achieve language enhancement. Electrophysiological methods, such as intracranially recorded EEG or ERP, are particularly promising to offer a mechanistic understanding of language formation processes. Compared with the language scales alone, the combination of in vivo measures of brain alterations in this study will be more sensitive in detecting acupuncture efficacy [50].

Therefore, this study can provide clinical evidence on the long-term efficacy of our acupuncture programme in patients with PSA and explore biomarkers for the recovery of function and the efficacy of acupuncture in patients with PSA through a multidimensional evaluation. This trial will fill the gaps in the evidence on the long-term efficacy of acupuncture for aphasia and provide a model for the multimodal evaluation of PSA.

## Figures and Tables

**Table 1:** Study design schedule.

Period	Screening and baseline	Treatment (w1–4)				Follow-up (w5–12)	Follow-up (w13–24)
Week (W)	W −1	W1	W2	W3	W4	W12	W24
Eligibility	×						
CT or MRI	×						
General information	×						
Physical examination	×				×	×	×
Medical history and demography	×						
Informed consent	×						
WAB		×			×	×	×
BDAE		×			×	×	×
Disease Prognosis Scale of ischaemic stroke		×			×	×	×
MRI (3D-T1, RS-fMRI, Task-fMRI, DTI)		×			×	×	×
EEG		×			×	×	×
ERP		×			×	×	×
Discomfort and acceptance of acupuncture		×	×	×	×	×	×
Assessment of blind method		×					
Adverse event			×	×	×	×	×
Compliance			×	×	×	×	×

CT, computed tomography; MRI, magnetic resonance imaging; WAB, Western Aphasia Battery; BDAE: Boston Diagnostic Aphasia Examination; RS-fMRI, resting-state functional magnetic resonance imaging; DTI, diffusion tensor imaging; EEG, electroencephalogram; ERP, event-related potentials.

**Table 2:** List of participating institutions and level of the institution.

Participating centre	Level of the institution
Dongzhimen Hospital Affiliated to Beijing University of Chinese Medicine	Tertiary A hospital
China Rehabilitation Research Center	Tertiary A hospital
Peking University Third Hospital	Tertiary A hospital

**Table 3:** Revised standards for reporting intervention in clinical trials of acupuncture (STRICTA [39]]).

Item	Item criteria	Description
(1) Acupuncture rationale	(1a) Style of acupuncture	Traditional Chinese medicine therapy
	(1b) Reasoning for treatment provided, based on historical context, literature sources, and/or consensus methods, with references where appropriate	(i) Reasoning for treatment provided—based on historical context, literature sources, and traditional Chinese medicine (consensus) (ii) Reasoning for treatment provided—based on historical context, literature [27,28], selection of treatment regions based on related papers, expert experience, and textbooks
	(1c) Extent to which treatment varied	Standardized treatment
(2) Details of needling	(2a) Number of needle insertions per subject per session (mean and range where relevant)	10 or 12
	(2b) Names (or location if no standard name) of points used (unilateral/bilateral)	DU20 (Baihui), EX-HN1 (Sishencong), HT5 (Tongli), GB39 (Xuanzhong), EX-HN12 (Jinjin), EX-HN13 (Yuye), CV23 (Lianquan)
	(2c) Depth of insertion, based on a specified unit of measurement or a particular tissue level	Needle insertion will follow an angle of 90°in an inferomedial direction for the two points (HT5, GB39). Depth: 0.5 cun [≈10 mm]. Needle insertion followed an angle of 15° in an inferomedial direction for the two points (DU20, EX-HN1). Depth: 0.5 cun. For RN23, the angle is 90° and the depth is 1 cun [≈20 mm]. EX-HN12 and EX-HN13 were quickly inserted for bloodletting
	(2d) Responses sought	Following needle insertion, small, equal manipulations of twirling and thrusting will be performed on all needles to reach de qi
	(2e) Needle stimulation	Small, equal manipulations of twirling and thrusting will be performed on the needles of HT5 and GB39
	(2f) Needle retention time	30 min per session
	(2g) Needle type	Sterile, stainless, disposable acupuncture needles (size 0.25 mm × 40 mm, product no. 20182270011; ANDE Acupuncture, Guizhou ANDE Medical Equipment, China)
(3) Treatment regimen	(3a) Number of treatment sessions	12
	(3b) Frequency and duration of treatment sessions	3 times/week, 30 min per session, on alternate days, for 4 successive weeks
(4) Other components of treatment	(4a) Details of other interventions administered to the acupuncture group	None
	(4b) Setting and context of treatment, including instructions to practitioners, and information and explanations to patients	The study will be conducted in the Dongzhimen Hospital affiliated to BUCM, China Rehabilitation Research Center, and Peking University Third Hospital, and all information will be provided to the subjects
(5) Practitioner background	(5a) Description of participating acupuncturists	The chief physician of Dongzhimen Hospital, Ph.D., 11 years of formal university training in traditional Chinese medicine, with qualifications for practising doctors stipulated in the law
(6) Control or comparator interventions	(6a) rationale for the control or comparator in the context of the research question, with sources that justify the choice	The nonacupoint control group will receive sham acupuncture with real acupuncture needles at nonacupoint locations. Through such an approach, the self-perception of placebo effects in the nonacupoint control group is difficult to distinguish from the real acupoints
	(6b) Precise description of the control or comparator; details for items 1–3 with the use of sham acupuncture or any other type of acupuncture-like control	Participants in the nonacupoint control group received sham acupuncture with a pragmatic placebo needle on a sham acupoint. The needles used are the same as the acupoint group. Procedures and other treatment settings will be the same as in the acupoint group but with no needle manipulation for de qi

**Table 4:** Location of acupoints used in the acupuncture group.

Acupoints	Location
DU20 (Baihui)	On the median line of the head, 5 cun superior to the anterior hairline, at about the middle on the connecting line between the two auricular tips
EX-HN1 (Sishencong)	On the vertex, 1 cun from the front, back, left, and right to DU20 (Baihui), common 4 points
HT5 (Tongli)	On the palmar side of the forearm, 1 cun superior to the transverse crease of the wrist, at the radial border of the ulnar carpal flexor muscular tendon
GB39 (Xuanzhong)	On the lateral side of the leg, 3 cun directly above the tip of lateral malleolus at the anterior border of the fibula
EX-HN12 (Jinjin)	In the mouth, EX-HN12 (Jinjin) is located with tongue furled, on the vein on the left side of the frenulum of the tongue
EX-HN13 (Yuye)	EX-HN13 (Yuye) is located on the vein on the right side of the frenulum of the tongue
CV23 (Lianquan)	In the neck, on the anterior midline, above the laryngeal protuberance, in the depression of the superior border of the hyoid bone

**Table 5:** Location of sham acupoints used in the NA group.

Nonacupoint (NA)	Location
NA 1	0.5 cun lateral to HT5 (Tongli)
NA 2	0.5 cun horizontal to GB39 (Xuanzhong)
NA 3	0.5 cun lateral to ST8 (Touwei)

## Data Availability

All data are available from the corresponding author upon reasonable request.

## Abbreviations

PSA: Poststroke aphasia
AQ: Aphasia quotient
WAB: Western Aphasia Battery
BDAE: Boston Diagnostic Aphasia Examination
MRI: Magnetic resonance imaging
EEG: Electroencephalogram
SLT: Speech and language therapy
TCM: Traditional chinese medicine
RCT: Randomized controlled trial
SPIRIT: Standard Protocol Items: Recommendations for Interventional Trials
CONSORT: Consolidated Standards of Reporting Trials
BUCM: Beijing University of Chinese Medicine
CT: Computed tomography
STRICTA: Standards for Reporting Interventions in Clinical Trials of Acupuncture
NA: Nonacupoint
RS-fMRI: Resting-state functional magnetic resonance imaging
DTI: Diffusion tensor imaging
ERP: Event-related potentials.

## Supplementary Materials

Supplementary Materials SPIRIT 2013 checklist: recommended items to address in a clinical trial protocol and related documents.
